# Comments to pharmacological and behavioral divergence of ketamine enantiomers by Jordi Bonaventura et al.

**DOI:** 10.1038/s41380-022-01447-4

**Published:** 2022-02-17

**Authors:** Guang Chen, Geert Mannens, Marlies De Boeck, Ella J. Daly, Carla M. Canuso, Greet Teuns, Husseini Manji, Wayne C. Drevets

**Affiliations:** 1 Neuroscience, Janssen Research & Development, LLC, San Diego, CA USA; 2 Drug Metabolism and Pharmacokinetics, Janssen Research & Development, Beerse, Belgium; 3 Non-Clinical Safety, Janssen Research & Development, Beerse, Belgium; 4 Neuroscience Medical Affairs, Janssen Scientific Affairs, LLC, Titusville, NJ USA; 5 Neuroscience Clinical Development, Janssen Research & Development, LLC, Titusville, NJ USA; 6 Science for Minds, Johnson & Johnson, New Brunswick, NJ USA

**Keywords:** Neuroscience, Molecular biology

## To the Editor:

We read with interest the article by Bonaventura et al. [1], who investigated the pharmacological properties of subanesthetic doses of ketamine enantiomers in rats and compared each enantiomer using rodent models of abuse liability. Consistent with the literature [2], the receptor affinity data of Bonaventura (Figure 1 [1]) showed that ketamine enantiomers are more potent for antagonizing N-methyl-D-aspartate receptors (NMDAR) than for activating mu-opioid receptors (MOR) and that (S)-ketamine is more potent than (R)-ketamine on both NMDAR and MOR. However, key elements of the study design limit the translation of their results to antidepressant treatment in humans (the only approved clinical indication for ketamine or its enantiomers in the *subanesthetic* dose range is the approved use of (S)-ketamine for reducing depressive symptoms in patients who manifest either treatment-resistant depression or major depressive disorder with acute suicidal ideation or behavior), and thus do not support some of the translational inferences which Bonaventura et al. draw from their data (see especially the “Clinical Implications” section of their Discussion). Most of the experiments conducted by Bonaventura et al. did not allow comparison between enantiomers at concentrations or doses that would be pharmacologically equipotent for antagonizing NMDAR receptors (see below) or for producing antidepressant effects in humans. The doses of (S)-ketamine tested in their rodent models (5–20 mg/kg administered by single IP injection) exceed the human antidepressant doses of ketamine (0.5 mg/kg, IV infused over 40 min) or (S)-ketamine (0.2 or 0.25 mg/kg, IV infused over 40 min) [2]. Based on reported mouse [3] and human [4] plasma drug level data, these doses would have resulted in plasma levels substantially higher than those reached during antidepressant treatment in humans.

Moreover, the dose range at which racemic ketamine produces anesthetic, analgesic, or antidepressant efficacy differs across these indications, and in each case the corresponding dose range for (S)-ketamine is lower than for ketamine [2]. The antidepressant dose-response relationship appears curvilinear for ketamine and (S)-ketamine, with optimal efficacy at ~0.5 mg/kg IV [5] and ~0.2 mg/kg IV [6], respectively. Results from a randomized, double-blind study comparing ketamine (0.5 mg/kg) and (S)-esketamine (0.25 mg/kg) in treatment-resistant depression [7] further confirmed that a dose ratio of ~2:1 for ketamine versus (S)-ketamine achieves comparable antidepressant effects. The antidepressant efficacy of (R)-ketamine and the efficacy comparison of (R)-ketamine with ketamine or (S)-ketamine have not been assessed in a randomized, controlled trial (RCT).

We previously reported that the estimated brain unbound levels of ketamine (0.5 mg/kg, IV infusion) and (S)-ketamine (84 mg, nasal spray) at their plasma C_max_ are 1 and 0.4 μM, respectively [8]. The reported K_i_-values of ketamine, (S)-ketamine, and (R)-ketamine for NMDAR are ~1, 0.5, and 2 μM, respectively [2]. The clinical antidepressant dose ratio of ~2:1 for ketamine versus (S)-ketamine is consistent with the ~2 fold higher K_i_ value for NMDAR of ketamine versus (S)-ketamine [2]. Notably, the K_i_-values of ketamine, (S)-ketamine, and (R)-ketamine for human MOR are 42.1, 28.6, and 83.8 μM, respectively [9], so the clinical antidepressant dose ratio is also consistent with differences in the K_i_-values of ketamine and (S)-ketamine for MOR. Nevertheless, the CNS side effects in humans (see below) and the difference between the estimated brain unbound drug level and K_i_-values for MOR do not support pharmacodynamically meaningful engagement of MOR at antidepressant doses of ketamine and (S)-ketamine in humans. Given the NMDAR or MOR potency differences and the known antidepressant dose ratio of ketamine versus (S)-ketamine, the dose at which (R)-ketamine would be expected to produce antidepressant efficacy would be higher than for (S)-ketamine, in proportion to the ratio of their potencies for NMDAR (4-fold [2] to 6-fold [1]). Such a dose ratio must also be considered when contemplating potential clinical implications of these enantiomers’ relative potencies for MOR (approximately threefold based on the K_i_ [9] or EC_50_ [1] data).

Therefore, while the authors concluded: “racemic ketamine’s abuse liability in humans is primarily due to the pharmacological effects of its (S)-enantiomer”, this statement overlooked the likelihood that if the (R)-enantiomer was used to treat depression, achieving an antidepressant effect putatively would depend on increasing the dose to achieve equipotency for NMDAR antagonism (which would also be the case if MOR stimulation played a role in the antidepressant mechanism), and that at such doses (R)-ketamine would show comparable abuse liability [10]. This point is supported by the one set of rodent abuse liability data reported by Bonaventura [1] that allowed comparison of doses equipotent for NMDAR antagonism: the pharmacodynamic effect on locomotor activity proved comparable between (S)-ketamine at 5 mg/kg IP and (R)-ketamine at 20 mg/kg IP (Figure 4 A of [1]). Crucially, the human literature also shows that the abuse liability of (S)- and (R)-ketamine, as indexed by dissociation and other CNS adverse effects, are dose dependent and when the doses being compared for each enantiomer are set at equipotency for NMDAR antagonism, their abuse potential appears similar (e.g., Oye et al. [10]).

Furthermore, Bonaventura [1] used 5–20 mg/kg of (S)-ketamine doses IP in their PET, locomotor activity, and conditioned place preference studies. As mentioned earlier, these doses would have resulted in plasma levels substantially higher than those reached during antidepressant treatment in humans, along with greater NMDAR blockade and engagement of MOR and other targets. The higher exposures tested by Bonaventura [1] are particularly relevant for interpreting the functional studies of MOR engagement. Bonaventura [1] demonstrated functional engagement of MOR at 10 μM of (S)-ketamine using in vitro functional screening and [^35^S]GTPγS autoradiography assays. In contrast, the estimated brain unbound concentration of (S)-ketamine at antidepressant doses in humans is ~0.4 μM [8], which is ~18 or ~70 fold lower than the reported K_i_-values of S-ketamine for MOR in rats (7 or 11 μM [1, 2]) or humans (28 μM [9]), respectively, suggesting that pharmacodynamically relevant, direct MOR engagement is unlikely to occur at human antidepressant doses. This inference is consistent with the absence of adverse events signifying MOR engagement (e.g., respiratory depression) in RCTs of patients with depression receiving (S)-ketamine [11], the absence of withdrawal effects observed following discontinuation of (S)-ketamine [12], and the absence of an effect of functional MOR gene polymorphisms on (S)-ketamine’s antidepressant effect [8]. Moreover, while disagreement exists within the literature as to whether MOR antagonism attenuates the antidepressant effects of racemic ketamine in humans [8], the MOR-preferring antagonist naltrexone does not block ketamine-induced dissociation [13].

Bonaventura [1] made other potentially misleading statements that merit comment. First, they drew a parallel between self-administration of (S)-ketamine IV by rats in an abuse-related behavioral paradigm to self-administration of (S)-ketamine nasal spray (Spravato^®^) by patients receiving antidepressant treatment. In the United States, Spravato^®^ is a CIII medication with restricted distribution, administered intermittently at doses consistent with the US product label, only under supervision by a healthcare professional in a certified healthcare setting, as part of a Risk Evaluation and Mitigation Strategy (REMS) program [14]. Notably, the data collected through REMs, pharmacovigilance, suspicious order monitoring, and other sources have shown no trends in abuse, misuse, or diversion [15].

In addition, the authors reference a study in which (R)-ketamine (0.5 mg/kg, IV) was administered *open-label* to seven patients with depression [16], characterizing these results as showing “antidepressant efficacy without dissociative effects”. Open-label study results cannot be used to establish efficacy, partly because they increase the expectancy biases of both the rater and the patient, and even patients with treatment-resistant depression have shown large placebo effect sizes in trials involving various treatment modalities [17]. Thus, as indicated by Bonaventura et al., the antidepressant efficacy and optimal dose of (R)-ketamine will need to be established in RCTs. Regarding dissociation, one of these patients manifested clinically meaningful dissociation, as indicated by a Clinician-Administered Dissociative States Scale score of 5. As mentioned above, a study in healthy volunteers showed that (R)-ketamine and (S)-ketamine produced dissociative and other CNS adverse effects that were similar in frequency, severity, and character when compared at doses selected to be equipotent for NMDAR antagonism [10].

In conclusion, the clinical inference of Bonaventura [1] that (R)-ketamine is unlikely to share the abuse liability of ketamine and (S)-ketamine is not justified by their study designs and results. We caution against drawing translational interpretations from these data without considering the limitations of the study designs.
